# Management of oral stomatitis associated with BRAFi-MEKi therapy using intralesional corticosteroid and local anesthetic: a case report

**DOI:** 10.31744/einstein_journal/2026RC2409

**Published:** 2026-08-21

**Authors:** Maria Cecilia Querido de Oliveira, Maria Cláudia Cuzzullin, Vallentine Louzada Miranda, Aline Paim de Abreu Paulo Gomes, Julio Cesar Marcassa, Pablo Augustin Vargas, Ana Luiza Oliveira Corrêa Roza, André Caroli Rocha, Alan Roger Santos-Silva, Ana Carolina Prado-Ribeiro, João Gabriel Silva Souza, Aljomar José Vechiato-Filho

**Affiliations:** 1 Universidade de São Paulo Faculdade de Medicina Instituto do Câncer do Estado de São Paulo São Paulo SP Brazil Dental Oncology Service, Instituto do Câncer do Estado de São Paulo, Faculdade de Medicina, Universidade de São Paulo, São Paulo, SP, Brazil; 2 Hospital Israelita Albert Einstein Faculdade Israelita de Ciências da Saúde Albert Einstein São Paulo SP Brazil Faculdade Israelita de Ciências da Saúde Albert Einstein, Hospital Israelita Albert Einstein, São Paulo, SP Brazil; 3 Universidade Guarulhos Dental Research Division Department of Periodontology Guarulhos SP Brazil Department of Periodontology, Dental Research Division, Universidade Guarulhos, Guarulhos, SP Brazil; 4 Centro Paulista de Radioterapia e Oncologia São Paulo SP Brazil Centro Paulista de Radioterapia e Oncologia, São Paulo, SP, Brazil; 5 Universidade Estadual de Campinas Faculdade de Odontologia de Piracicaba Department of Oral Diagnosis Piracicaba SP Brazil Oral Pathology Section, Department of Oral Diagnosis, Faculdade de Odontologia de Piracicaba, Universidade Estadual de Campinas, Piracicaba, SP Brazil; 6 Texas A&M University College of Dentistry Department of Diagnostic Sciences Dallas TX United States Department of Diagnostic Sciences, Texas A&M University College of Dentistry, Dallas, TX, United States; 7 Universidade de São Paulo Hospital das Clínicas Department of Oral and Maxillofacial Surgery São Paulo SP Brazil Department of Oral and Maxillofacial Surgery, Hospital das Clínicas, Universidade de São Paulo, São Paulo, SP Brazil; 8 Faculdade de Ciências Odontológicas Dental Science School Montes Claros MG Brazil Dental Science School, Faculdade de Ciências Odontológicas, Montes Claros, MG, Brazil

**Keywords:** BRAF inhibitor, MEK inhibitor, Mouth diseases, Dabrafenib, Melanoma, trametinib, Adrenal cortex hormones

## Abstract

This case documents the clinical pattern of a fibrinopurulent ulcer with a hyperplastic lesion that developed after the initiation of two classes of targeted therapies acting on different components of the mitogen-activated protein kinase signaling pathway (BRAF inhibitors [BRAFi] and MEK inhibitors [MEKi]). A 66-year-old woman was diagnosed with choroidal melanoma in the right eye in 2012 and successfully treated by choroidal melanoma resection. Tumor recurrence was diagnosed 8 years later. Nivolumab and ipilimumab were prescribed, but this combination was replaced with dabrafenib-trametinib due to disease progression. A second orbital exenteration and adjuvant radiotherapy to the orbital cavity (total dose of 4,000 cGy) were performed during dabrafenib-trametinib therapy. A few months after initiating this combination, the patient reported oral discomfort and an ulcer on the dorsum of the tongue. An incisional biopsy showed only signs of inflammation. A second biopsy demonstrated the same histological pattern. Based on clinical and histopathological findings, the final diagnosis was stomatitis associated with BRAFi-MEKi therapy. An intralesional injection of triamcinolone solution combined with 2% mepivacaine was performed. One month later, 0.05% topical corticosteroid was prescribed, and the lesion completely healed after an additional month. This report describes a previously unreported clinical presentation of an oral adverse event associated with combined BRAFi-MEKi therapy. It highlights the importance of recognizing unusual oral toxicities related to targeted therapies, as well as the role of appropriate local management in controlling symptoms and allowing the continuation of oncologic treatment.

## INTRODUCTION

Targeted therapies and immunotherapies have been developed to improve patient prognoses by enhancing the efficiency of cancer treatment and reducing its side effects.(1,2,3) These therapies induce apoptosis in cancer cells and reduce their proliferation by blocking specific cellular pathways related to carcinogenesis and tumor growth. Additionally, these treatments may correct certain "flaws" in an individual's immune system, enhancing its ability to target and kill cancer cells.(1,2,3)

It is estimated that 324,635 people were diagnosed with melanoma skin cancer worldwide in 2020, and 57,043 died of the disease.(4) However, the field of melanoma therapy for advanced and/or metastatic lesions has progressively improved in recent years. Further understanding of the molecular mechanisms related to cellular proliferation and growth has resulted in the development of B-Raf proto-oncogene serine/ threonine-protein kinase (BRAF) and mitogen-activated protein kinase (MEK) inhibitors for melanoma treatment. These two classes of targeted therapies act on different components of the mitogen-activated protein kinase (MAPK) signaling pathway.(5) In addition, improvements in the ability to regulate T-cell activation by blocking specific immune checkpoints have led to the development of immune checkpoint inhibitors (ICIs) for the treatment of melanoma. These include antibodies against cytotoxic T lymphocyte-associated protein 4 (CTLA-4), programmed death-1 (PD-1), and its ligand, programmed death-ligand 1 (PD-L1).(5)

Despite the expectation that targeted therapies and immunotherapies would induce fewer adverse events (AEs) than systemic chemotherapy, specific AEs have been reported.(3,5) Cutaneous toxicity has been observed in 92%-99% of patients receiving treatment with BRAF inhibitors (BRAFi).(6) Dabrafenib, a BRAFi, has been associated with cutaneous and oral hyperkeratotic reactions, which may develop within the first few weeks of treatment.(2,6,7) Fortunately, the combination with MEK inhibitors (MEKi), such as trametinib, appears to reduce these undesired side effects.(2,8) AEs have also been reported with ICI therapies, such as nivolumab or ipilimumab.(9,10,11)

Oral adverse events (OAEs) resulting from targeted therapies and ICIs are a matter of concern due to their negative impact on patients' ability to tolerate cancer treatment, the increased financial burden, and the reduction in quality of life. (3,12,13,14,15,16) Furthermore, it is important that dentists, oncologists, and patients are aware of documented OAEs, as well as the possibility that previously unreported events may occur as these therapies become more widely used in oncology. Such awareness is important because OAEs may necessitate discontinuation or interruption of treatment if oral lesions are not adequately managed. (3,17,18,19)

Considering that impaired oral health may interrupt oncologic treatment continuity, particularly when systemic conditions are negatively affected by cancer or toxicities resulting from therapeutic regimens, the management of OAEs is a key factor in appropriate health care.(12,13,20) Up to 90% of patients undergoing certain targeted therapies may develop such conditions. Therefore, these manifestations represent a notable barrier to oncology treatment continuity, whereby unplanned treatment breaks directly compromise the dose intensity required to suppress tumor progression and may reduce overall survival rates by up to 20%.(16) Additionally, evidence suggests that the absence of proper dental care can increase the complexity of dental treatment, as well as systemic complications and related healthcare costs.(20)

The limited number of studies in the literature associating oral reactions with the aforementioned medications highlights the importance of the clinical case presented herein. In this case, a patient treated with a BRAFi (dabrafenib) developed a fibrinopurulent ulcer with a hyperplastic-like lesion, despite concurrent treatment with a MEKi (trametinib). In this context, a recent narrative review described adverse reactions in the oral cavity as lesions with the clinical features of oral mucositis (ulcers associated with erythematous areas of the mucosa), which are very similar to the clinical presentation of stomatitis related to other epithelial growth factor receptor inhibitors.(3,21) Therefore, to the best of our knowledge and based on a focused literature review of oral adverse events associated with BRAFi and MEKi therapy, no previous reports describing a fibrinopurulent ulcer with hyperplastic-like oral lesions in patients receiving combined BRAFi-MEKi therapy have been identified. The lesion was successfully treated with intralesional and topical corticosteroids.

## CASE REPORT

A 66-year-old woman was diagnosed with choroidal melanoma in her right eye in 2012. The patient underwent choroidal melanoma resection, which successfully controlled the disease for eight years. However, in 2020, an orbital melanoma lesion was identified, and a metastatic tumor in the liver was diagnosed during routine examinations. The oncologist modified the treatment approach and initiated combination therapy with nivolumab and ipilimumab, which was maintained until 2022. Subsequently, further modification to the patient's cancer treatment was necessary to improve disease control and prognosis. Dabrafenib and trametinib were prescribed, and orbital exenteration was performed. Following this, the patient underwent adjuvant radiotherapy to the orbital cavity, administered in 16 fractions of 250cGy each, resulting in a total radiation dose of 4,000cGy.

A few months after initiating this combination, the patient reported oral discomfort and an ulcer on the dorsum of the tongue. As a result, the oncologist interrupted treatment pending diagnosis of the oral lesion.

The tongue lesion persisted, and a head and neck surgeon performed an incisional biopsy, which showed only signs of inflammation (nonspecific chronic glossitis). The patient was subsequently referred to a dental office for photobiomodulation using low-level laser therapy to promote healing and analgesia. However, no satisfactory results were obtained. Detailed information regarding the low-level laser therapy parameters is unavailable, as the procedure had been performed by another professional.

The patient was then referred to a stomatologist in private practice, and a new biopsy of a fibrinopurulent ulcer with a hyperplastic-like lesion was performed in two different areas and submitted to an oral pathologist for specialized analysis (Figure 1). The results showed the same histological pattern as the first biopsy, with no evidence of malignant cells, and the initial descriptive diagnosis was maintained (Figure 2).

**Figure 1:**
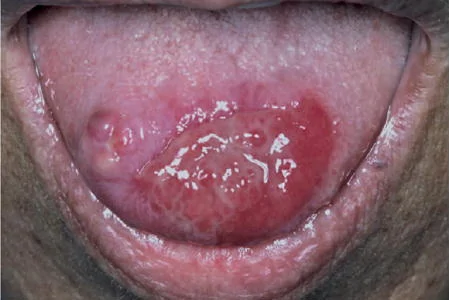
Initial appearance of the oral lesion (before the second incisional biopsy)

**Figure 2:**
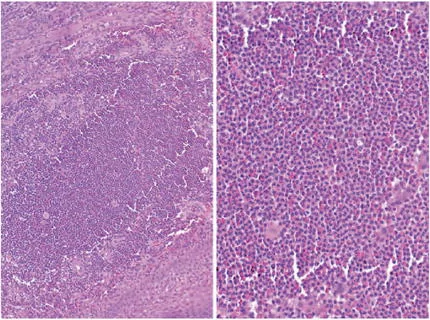
Histological examination of tongue biopsy specimens revealed oral mucosa with pseudoepitheliomatous hyperplasia and extensive ulceration, a dense lymphoplasmacytic inflammatory infiltrate, focal abscess formation, and scattered eosinophils. No epithelial dysplasia or malignancy was identified. S0X10 immunohistochemistry was negative, excluding melanocytic differentiation

Considering all information collected during anamnesis, the patient's clinical course, hemogram results, and discussions with their oncologist and radiation oncologist, BRAFi-MEKi-associated stomatitis was deemed the most likely diagnosis. Therefore, an intralesional injection consisting of triamcinolone acetonide (20mg/mL) mixed with 2% mepivacaine in a 1:1 ratio, for a total volume of approximately 1mL, was administered directly into the lesion. One month after this treatment, the lesion was significantly reduced but not completely healed (Figure 3). Subsequently, 0.05% topical clobetasol propionate was prescribed twice daily for one month, after which the lesion completely healed (Figure 4). No recurrence was observed during a 4-month follow-up period.

**Figure 3:**
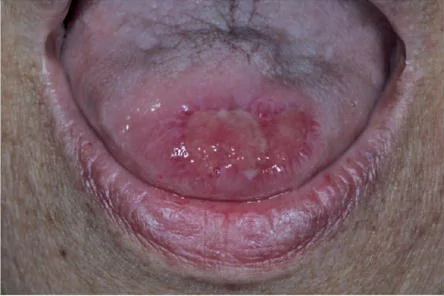
Clinical appearance of the oral lesion 1 month after intralesional injection of triamcinolone and 2% mepivacaine (1:1)

**Figure 4:**
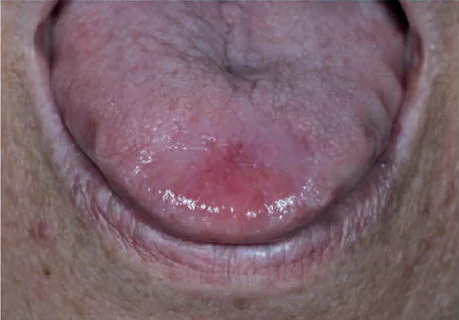
Final clinical appearance of the oral lesion 1 month after twice-daily application of 0.05% topical clobetasol propionate, showing complete healing

## ETHICAL APPROVAL

This study was performed in accordance with the Helsinki Declaration, and written informed consent was obtained. This article was approved by the Research Ethics Committee of the *Faculdade de Odontologia de Piracicaba, Universidade Estadual de Campinas* (FOP/UNICAMP), CAAE: 98530526.5.0000.5418; #8.623.820.

## DISCUSSION

Choroidal melanoma is the most common primary intraocular malignant tumor. This condition is generally diagnosed in patients with a median age of 55 years and is slightly more frequent in men.(22,23) The liver is the most common site for metastatic lesions (approximately 90%) derived from choroidal melanoma.(24,25) In the present case, only the patient's sex diverged from this typical disease pattern.

Immune checkpoint inhibitors have different targets of action within the patient's immune system, leading to a range of manifestations and toxicities. For example, oral adverse events have been reported with the use of PD-1/PD-L1-targeted ICIs (e.g., nivolumab), including ulcerative or erythematous lesions.(26) In addition, approximately 20% of patients treated with BRAFi monotherapy may develop hyperproliferative lesions.(27,28) Furthermore, hyperkeratotic lesions have been reported in intraoral keratinized and non-keratinized areas in patients treated with BRAFi therapy alone.(7) In the present case, the patient developed a fibrinopurulent ulcer with a hyperplastic-like lesion on the tongue, which appears to combine features of OAEs associated with both ICI therapy and BRAFi therapy. Interestingly, the patient was receiving a MEKi (trametinib) in combination with a BRAFi (dabrafenib), which is generally thought to reduce the incidence of OAEs.(22,26,27,28,29)

Unlike mammalian target of rapamycin (mTOR) therapies,(18,19) OAEs associated with BRAFi and ICI therapies have only recently been described.(20,26,27,29,30) Establishing an association between oral lesions and targeted therapies can be challenging, as oncology patients often receive multiple, sometimes concomitant, treatments throughout their clinical course. In the present case, alternative etiologies were systematically considered and excluded. Radiation-induced mucosal toxicity was deemed unlikely, as radiotherapy was restricted to the orbital cavity, and no radiation field involved the oral mucosa, as confirmed by the radiotherapy treatment plan. Infectious etiologies were also considered but were not supported by clinical evolution or histopathological findings.

Oncologic lesions (metastatic or a new primary tumor) were excluded by two oral biopsies performed by different professionals (a head and neck surgeon and a stomatologist), both of which showed no evidence of malignancy. It is important to note that these laboratory analyses were conducted by two pathologists from different facilities. A traumatic origin was considered unlikely due to the lesion's location on the dorsum of the tongue and the absence of local irritative factors. Furthermore, immune-related mucosal toxicity from prior ICI therapy was considered less probable given the long interval between immunotherapy discontinuation and lesion onset. Therefore, the temporal association between the initiation of dabrafenib-trametinib therapy and lesion development, combined with the absence of alternative explanations, supports the diagnosis of BRAFi-MEKi-associated stomatitis as a "probable" adverse drug reaction according to pharmacovigilance principles, such as the Naranjo Adverse Drug Reaction Probability Scale.(31)

Current literature suggests that mucocutaneous adverse events associated with BRAF inhibitors may be related to alterations in the MAPK signaling pathway. Hyperkeratotic lesions have been attributed to the proliferation of wild-type BRAF keratinocytes, a phenomenon described as "paradoxical activation" of the MAPK pathway.(32) This pathway plays a central role in several cellular regulatory processes, including cell growth, proliferation, migration, cell cycle control, and apoptosis, and it is mediated through sequential phosphorylation of RAS, RAF, MEK, and ERK proteins.(33,34) In this context, modulation of the MAPK pathway by BRAF and MEK inhibitors may influence keratinocyte behavior, potentially leading to inflammatory changes, impaired cell migration, or cell death.(35,36) Given their high turnover rate, oral mucosal cells may be particularly susceptible to such alterations.(21) However, although MAPK pathway dysregulation has been proposed as a potential mechanism for mucocutaneous toxicities associated with these therapies, the exact biological basis of oral lesions remains incompletely understood. Therefore, the mechanistic interpretation presented here should be considered hypothetical rather than a definitive causal explanation.

In the present case, the patient developed a persistent intraoral lesion without evidence of malignancy on histopathological analysis. Although combined BRAFi-MEKi therapy is generally associated with a reduction in hyperproliferative reactions, the lesion did not regress during oncologic treatment. After other potential causes were considered, the lesion was interpreted as a possible oral adverse event associated with BRAFi-MEKi therapy. Management with intralesional and topical corticosteroids resulted in progressive clinical improvement, with complete healing of the lesion. This case highlights the importance of recognizing unusual oral toxicities related to targeted therapies, as well as the role of appropriate local management in controlling symptoms and allowing continuity of oncologic treatment.

## Data Availability

The data supporting this study's findings are available from the corresponding author upon reasonable request.

## References

[1] Joo WD, Visintin I, Mor G. (2013). Targeted cancer therapy—are the days of systemic chemotherapy numbered?. Maturitas.

[2] Lacouture M, Sibaud V. (2018). Toxic Side Effects of Targeted Therapies and Immunotherapies Affecting the Skin, Oral Mucosa, Hair, and Nails. Am J Clin Dermatol.

[3] Gomes-Silva W, Vechiato AJ, Luiz AC, Guollo A, de Oliveira MC, Gomes MN, (2023). Clinical characterization of stomatitis cases with an epithelial growth factor receptor inhibitor in metastatic colorectal cancer patients: A study of 7 cases and literature review. Oral Surg Oral Med Oral Pathol Oral Radiol.

[4] Sung H, Ferlay J, Siegel RL, Laversanne M, Soerjomataram I, Jemal A, (2021). Global Cancer Statistics 2020: GLOBOCAN Estimates of Incidence and Mortality Worldwide for 36 Cancers in 185 Countries. CA Cancer J Clin.

[5] de Golian E, Kwong BY, Swetter SM, Pugliese SB. (2016). Cutaneous Complications of Targeted Melanoma Therapy. Curr Treat Options Oncol.

[6] Lacouture ME, Duvic M, Hauschild A, Prieto VG, Robert C, Schadendorf D, (2013). Analysis of dermatologic events in vemurafenib-treated patients with melanoma. Oncologist.

[7] Carlos G, Anforth R, Clements A, Menzies AM, Carlino MS, Chou S, (2015). Cutaneous toxic effects of BRAF inhibitors alone and in combination with MEK inhibitors for metastatic melanoma. JAMA Dermatol.

[8] Belum VR, Marulanda K, Ensslin C, Gorcey L, Parikh T, Wu S, (2015). Alopecia in patients treated with molecularly targeted anticancer therapies. Ann Oncol.

[9] Hodi FS, O'Day SJ, McDermott DF, Weber RW, Sosman JA, Haanen JB, (2010). Improved survival with ipilimumab in patients with metastatic melanoma. N Engl J Med.

[10] Macdonald JB, Macdonald B, Golitz LE, LoRusso P, Sekulic A. (2015). Cutaneous adverse effects of targeted therapies: Part II: Inhibitors of intracellular molecular signaling pathways. J Am Acad Dermatol.

[11] Choi JN. (2014). Dermatologic adverse events to chemotherapeutic agents, Part 2: BRAF inhibitors, MEK inhibitors, and ipilimumab. Semin Cutan Med Surg.

[12] Kauark-Fontes E, Rodrigues-Oliveira L, Epstein JB, Faria KM, Araújo AL, Gueiros LA, (2021). Cost-effectiveness of photobiomodulation therapy for the prevention and management of cancer treatment toxicities: a systematic review. Support Care Cancer.

[13] Epstein JB, Thariat J, Bensadoun RJ, Barasch A, Murphy BA, Kolnick L, (2012). Oral complications of cancer and cancer therapy: from cancer treatment to survivorship. CA Cancer J Clin.

[14] Lalla RV, Saunders DP, Peterson DE. (2014). Chemotherapy or radiation-induced oral mucositis. Dent Clin North Am.

[15] Jehn P Stier R, Tavassol F, Dittmann J, Zimmerer R, Gellrich NC, (2019). Physical and Psychological Impairments Associated with Mucositis after Oral Cancer Treatment and Their Impact on Quality of Life. Oncol Res Treat.

[16] Rosenthal DI. (2007). Consequences of mucositis-induced treatment breaks and dose reductions on head and neck cancer treatment outcomes. J Support Oncol.

[17] Sonis S, Treister N, Chawla S, Demetri G, Haluska F. (2010). Preliminary characterization of oral lesions associated with inhibitors of mammalian target of rapamycin in cancer patients. Cancer.

[18] Lo Muzio L, Arena C, Troiano G, Villa A. (2018). Oral stomatitis and mTOR inhibitors: A review of current evidence in 20,915 patients. Oral Dis.

[19] Shameem R, Lacouture M, Wu S. (2015). Incidence and risk of high-grade stomatitis with mTOR inhibitors in cancer patients. Cancer Invest.

[20] Vigarios E, Lamant L, Delord JP, Fricain JC, Chevreau C, Barrés B, (2015). Oral squamous cell carcinoma and hyperkeratotic lesions with BRAF inhibitors. Br J Dermatol.

[21] Basilicata M, Terrano V, D'Aurelio A, Bruno G, Troiani T, Bollero P (2024). Oral Adverse Events Associated with BRAF and MEK Inhibitors in Melanoma Treatment: A Narrative Literature Review. Healthcare (Basel).

[22] Albert DM., Albert DM, Jakobiec FA (1994). Principles and Practice of Ophthalmology.

[23] Jensen OA. (1963). Malignant melanoma of the uvea in Denmark 1943-1952, a clinical histopathological, and prognostic study. Acta Ophthalmol Suppl (Copenh).

[24] Diener-West M, Reynolds SM, Agugliaro DJ, Caldwell R, Cumming K, Earle JD, Green DL, Hawkins BS, Hayman J, Jaiyesimi I, Kirkwood JM, Koh WJ, Robertson DM, Shaw JM, Thoma J (2004). Collaborative Ocular Melanoma Study Group Report 23. Screening for metastasis from choroidal melanoma: the Collaborative Ocular Melanoma Study Group Report 23. J Clin Oncol.

[25] Singh P, Singh A. (2012). Choroidal melanoma. Oman J Ophthalmol.

[26] Sibaud V, Eid C, Belum VR, Combemale P, Barres B, Lamant L, (2017). Oral lichenoid reactions associated with anti-PD-1/PD-L1 therapies: clinicopathological findings. J Eur Acad Dermatol Venereol.

[27] Flaherty KT, Infante JR, Daud A, Gonzalez R, Kefford RF, Sosman J, (2012). Combined BRAF and MEK inhibition in melanoma with BRAF V600 mutations. N Engl J Med.

[28] Chapman PB, Hauschild A, Robert C, Haanen JB, Ascierto P, Larkin J, Dummer R, Garbe C, Testori A, Maio M, Hogg D, Lorigan P, Lebbe C, Jouary T, Schadendorf D, Ribas A, O'Day SJ, Sosman JA, Kirkwood JM, Eggermont AM, Dreno B, Nolop K, Li J, Nelson B, Hou J, Lee RJ, Flaherty KT, McArthur GA, BRIM-3 Study Group (2011). Improved survival with vemurafenib in melanoma with BRAF V600E mutation. N Engl J Med.

[29] Larkin J, Ascierto PA, Dréno B, Atkinson V, Liszkay G, Maio M, (2014). Combined vemurafenib and cobimetinib in BRAF-mutated melanoma. N Engl J Med.

[30] Vigarios E, Epstein JB, Sibaud V. (2017). Oral mucosal changes induced by anticancer targeted therapies and immune checkpoint inhibitors. Support Care Cancer.

[31] Naranjo CA, Busto U, Sellers EM, Sandor P Ruiz I, Roberts EA, (1981). A method for estimating the probability of adverse drug reactions. Clin Pharmacol Ther.

[32] Chu EY, Wanat KA, Miller CJ, Amaravadi RK, Fecher LA, Brose MS, (2012). Diverse cutaneous side effects associated with BRAF inhibitor therapy: a clinicopathologic study. J Am Acad Dermatol.

[33] Robinson MJ, Cobb MH. (1997). Mitogen-activated protein kinase pathways. Curr Opin Cell Biol.

[34] McCubrey JA, Steelman LS, Chappell WH, Abrams SL, Wong EW, Chang F, (2007). Roles of the Raf/MEK/ERK pathway in cell growth, malignant transformation and drug resistance. Biochim Biophys Acta.

[35] Manousaridis I, Mavridou S, Goerdt S, Leverkus M, Utikal J. (2013). Cutaneous side effects of inhibitors of the RAS/RAF/MEK/ERK signalling pathway and their management. J Eur Acad Dermatol Venereol.

[36] Sinha R, Edmonds K, Newton-Bishop JA, Gore ME, Larkin J, Fearfield L. (2012). Cutaneous adverse events associated with vemurafenib in patients with metastatic melanoma: practical advice on diagnosis, prevention and management of the main treatment-related skin toxicities. Br J Dermatol.

